# Aqueous Extract of *Artemisia annua* Shows In Vitro Antimicrobial Activity and an In Vivo Chemopreventive Effect in a Small-Cell Lung Cancer Model

**DOI:** 10.3390/plants11233341

**Published:** 2022-12-01

**Authors:** Khaled S. Allemailem

**Affiliations:** Department of Medical Laboratories, College of Applied Medical Sciences, Qassim University, Buraydah 51452, Saudi Arabia; k.allemailem@qu.edu.sa; Tel.: +966-163010555

**Keywords:** *Artemisia annua*, aqueous extract, small-cell lung cancer (SCLC), chemoprevention

## Abstract

*Artemisia annua* (*A. annua*) has been used as a medicinal plant in the treatment of several infectious and non-infectious diseases in the forms of tea and press juice since ancient times. The aim of this study was to evaluate the aqueous extract of *A. annua* (AAE) as an antimicrobial agent in vitro and to evaluate its chemopreventive efficacy in vivo in a small-cell lung cancer (SCLC) animal model. The dried powder of AAE was prepared using the Soxhlet extraction system from the leaves of *Artemisia annua*. The in vitro activity of AAE was determined against *Candida albicans* (*C. albicans*), *Enterococcus faecalis* (*E. faecalis*), *Klebsiella pneumoniae* (*K. pneumoniae*), and methicillin-resistant *Staphylococcus aureus* (MRSA) using the agar well diffusion method and propidium iodide (PI)-stained microbial death under a confocal microscope. The pretreatment of mice with AAE was initiated two weeks before the first dose of benzo[a]pyrene and continued for 21 weeks. The chemopreventive potential of the extract was evaluated by flow cytometry and biochemical and histopathological analyses of the tissues and serum accordingly, after sacrificing the mice. The data revealed the antimicrobial potential of AAE against all the species investigated, as it showed growth-inhibitory activity by MIC, as well as confocal microscopy. The pretreatment of AAE exhibited significant protection in carcinogen-modulated, average body weight (ABW), and relative organ weight (ROW) cancer biomarkers in the serum and antioxidants in the lungs. The hematoxylin and eosin (H&E) staining of the tissues revealed that AAE prevented malignancy in the lungs. AAE also induced apoptosis and decreased intracellular reactive oxygen species (ROS) in the lung cells analyzed by flow cytometry. The current findings demonstrated the use of AAE as an alternative medicine in the treatment of infectious disease and the chemoprevention of lung cancer. To our knowledge, this is the first study that summarizes the chemopreventive potential of AAE in a lung cancer model in vivo. However, further investigations are suggested to understand the role of AAE to potentiate the therapeutic index of the commercially available drugs that show multiple drug resistance against microbial growth and high toxicity during cancer chemotherapy.

## 1. Introduction

The use of plant materials as nutraceuticals has made major contributions in the development of drugs against cancer and infectious diseases in the last two decades, as reviewed by several researchers [1,2,3,4,5,6]. The combination of more than one substance developing a greater sum than their separate effects is commonly known as synergy. Keeping the notion of nutraceuticals in consideration, the synergistic effects of many secondary metabolites have been evaluated, as reviewed in several publications [7,8,9,10]. A huge scientific literature focused on it, encompassing hundreds of thousands of scientific papers, has emerged in the current decade. However, the development of an effective combination for the treatments of the diseases is one of the most challenging areas of research.

The Identification of the active molecule in the herbal extracts is also an interesting task, as the extracts contain hundreds or sometimes thousands of constituents at varying concentrations. As shown in several reports, there are only a limited number of known constituents that are responsible for the overall activity of extracts [11,12]. However, various studies have been shown to have the synergistic, additive, or antagonistic effects by the mixture of different constituents in the extract, while the activity was lost following fractionation [11,12,13,14,15,16]. Thus, the use of crude extracts may have great potential, in comparison to the two different bioactive constituents.

Recently, *Artemisia annua* has engrossed widespread attention, as it has been used traditionally against several diseases, including cancer, for centuries [17,18]. Some of the studies revealed that *A. annua* is among one of the four plants that showed the highest ORAC (oxygen radical absorbance capacity) due to high phenolic content [19,20]. The dried leaf extracts of A. annua have been shown to have antibacterial, antifungal, and antileishmanial, as well as anticancer potential [21,22,23,24,25]. Furthermore, it played an important role in malaria-related mortality, as it has been adopted as the best remedy for the treatment of malaria in several developing countries [26,27,28]. Noticeably, the use of artemisinin, a major bioactive constituent of *A. annua*, has made the paradigm shift in antimalarial research and therapy and saved millions of lives. Professor Youyou, who discovered the artemisinin and tested its effects broadly against malaria, was awarded the Nobel Prize in Physiology or Medicine for her contribution [29,30]. Evidently, the data revealed its potential in the relief of pain, stiffness, and osteoarthritis-related complications [31,32]. Moreover, multiple studies have demonstrated the in vitro anticancer activity and the possible molecular mechanisms of *A. annua*, while very few studies explore its efficacy in animal models in detail [33,34,35,36]. Several studies also reported the efficacy of *A. annua* and its active constituent artemisinin against small-cell lung cancer (SCLC) in vitro [33,37,38], so there is a need to evaluate its potential in lung cancer systems in vivo. The consumption of tobacco is one the main culprits of lung cancer, as it escalates the progression of the disease several-fold [39,40,41,42]. As evident from various research, the BaP is the active component in the smoke of cigarettes and is metabolized into the more active BaP diol epoxide, which forms the DNA adduct, thus participating in the initiation, promotion, and progression of SCLC. Thus, the BaP is suggested to be a highly suitable carcinogen, which has been studied in all the events of lung carcinogenesis in rodents [43,44,45].

The aim of the present study is to evaluate the antimicrobial activity of *A. annua* in vitro as a primary screening. Our main objective is to investigate the chemopreventive potential of *A. annua* in a carcinogen-induced SCLC animal model.

## 2. Methods

### 2.1. Materials

The Benzo [a] Pyrene (BaP) was obtained from Sigma-Aldrich (St. Louis, MO, USA). The annexin V-FITC apoptosis assay kit was procured from Miltenyi Biotec (Bergisch Gladbach, Germany). 2′,7′-Dichlorofluorescin diacetate (ab273640), cancer markers, and antioxidant enzyme assay kits were purchased from Abcam (Cambridge, MA, USA). The leaves of *A. annua* were purchased from the regional pharmacy at Buraydah, Saudi Arabia. Other reagents and lab supplies were procured from local distributors.

### 2.2. Preparation of Aqueous Extract of Artemisia annua (AAE) from Leaves

The AAE was prepared using the Soxhlet extraction system from the leaves of *A. annua* following defatting with cyclohexane. The dried, coarsely ground leaves (100 g) were immersed in cyclohexane (300 mL) in the beaker with constant stirring for 4 h. The defatted powder was dried at 40 °C overnight following centrifugation of the mixture for 10 min at 5000 rpm; the supernatant containing cyclohexane, soluble lipids, and soluble cyclohexane was discarded. The dried powder was placed in a thimble for extraction in an automated Soxhlet extraction system (Buchi B-811, Flawil, Switzerland). The extraction was performed for several hours, and the distilled water was added as a solvent in the collecting beaker; finally, it was evaporated to get the crude, dried powder of *A. annua*.

### 2.3. Microorganisms

ATCC strains of *Candida albicans* (*C. albicans*): ATCC 60193; *Enterococcus faecalis* (*E. faecalis*): ATCC 29,212; methicillin-resistant *Staphylococcus aureus* (MRSA): ATCC 43,300; and *Klebsiella pneumoniae* (*K. pneumoniae*): ATCC 700,603 were procured from PGIMER, Chandigarh, India and used in the current study.

### 2.4. Determination of Antimicrobial Activity of AAE by the Well Diffusion and the Dilution Methods

The in vitro antimicrobial activity of AAE was determined using the agar well diffusion method as described in our earlier study [46]. *C. albicans*, *E. faecalis,* MRSA, and *K. pneumoniae* were plated on Mueller Hilton Agar plates. Holes of 8 mm in diameter were made with the help of a 1 mL pipette tip. The treating wells were filled with 50 µL of sterile PBS solutions containing 6 mg/mL (high concentration) and 3 mg/mL (low concentration) of AAE, while the control wells were loaded with 50 µL of sterile PBS. After 24 h of the incubation at 37 °C, the zone of growth inhibition was measured.

The minimum inhibitory concentration (MIC) of AAE was also determined using the macrodilution method as reported in an earlier study [46]. In order to determine the MIC of AAE, the concentrations of the extracts were taken from a range of 25 µg/mL to 5 mg/mL. An amount of 100 μL of bacterial or *C. albicans* suspension containing 1 × 10^5^ CFUs was inoculated in the test tubes that had 3 mL of Tryptic soya broth (TSB). The MIC of AAE was considered the lowest concentration of the extracts at which there was no visible growth of bacteria or *C. albicans*.

### 2.5. Analysis of the Microbial Death by the Confocal Microscopy

The above-mentioned bacteria (*E. faecalis, MRSA, and K. pneumonia*) *and C. albicans* (1 × 10^5^ CFUs) were cultured in TSB in the presence or absence of low-concentration (3 mg/mL) and high-concentration (6 mg/mL) AAE in 12-well sterile culture plates. After 24 h, the plates were gently washed with PBS. Bacterial and fungal cells were stained with propidium iodide (PI), then washed with PBS. The analysis of the cells was conducted under the confocal microscope using a 20X magnification objective as described earlier [46].

### 2.6. In Vivo Studies

#### 2.6.1. Mice

The experimental female Swiss albino mice, 8–10 weeks old, were obtained from the animal facility of King Saud University, Riyadh, Saudi Arabia. All the animal experiments, including the induction of lung cancer using chemical carcinogen, bleeding, and injection, as well as the sacrifice of the mice, were conducted following guidelines of the University of London Animal Welfare Society, Wheathampstead, England. The study protocol was approved by Animal Ethical Committee, Qassim University, Saudi Arabia, as QU-IF-02-03-27716. All the experimental animals were housed in the animal facility of CAMS as per the guidelines and monitored throughout the study twice a day by well trained and dedicated staff members. All the surviving animals were euthanized by CO_2_ inhalation at the end of the study following approved procedure. However, the mice were also euthanized in CO_2_ chamber within 2–4 h, whether they were moribund, or observed by a lack of sustained purposeful response to gentle stimuli. None of the mice died during the experiment before euthanasia.

#### 2.6.2. Experimental Design

**Figure 1:**
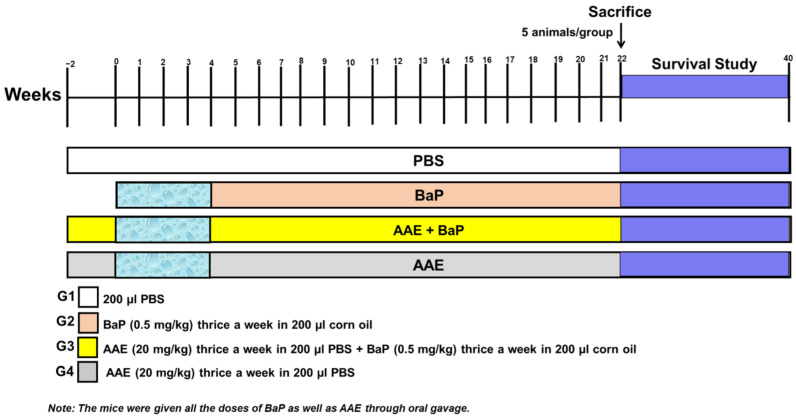
Experimental design of the in vivo studies. G1 (PBS) three times a week from −2 to week 21. G2 (BaP, 50 mg/kg b.w in 200 µL corn oil) thrice a week from week 0 to week 4. G3 (AAE 20 mg/kg in PBS) as G1 + BaP as G2, and G4 (AAE 20 mg/kg) as G1. All the animals were administered PBS and BaP, as well as AAE, orally.

The 60 mice were randomly allocated into four groups, with each group consisting of 15 animals. As illustrated in detail in Figure 1, the small-cell lung cancer (SCLC) was initiated and promoted by the exposure of 50 mg/kg body weight (b.w) BaP in 200 µL of corn oil, thrice a week for 4 weeks through oral gavage, as described earlier [47,48]. The AAE (20 mg/kg b.w) was started via oral gavage, two weeks before the first dose of BaP, and continued for 21 weeks. At the end of twenty-two weeks after the first dose of BaP, 5 animals from each group were sacrificed for further biochemical and histological analyses, as described earlier [47,48]. The mice were euthanized within 2–4 h when they showed no response, as stated above during the observational study, but they were reported dead in the survival data.

### 2.7. Investigation of Average Body Weight (ABW), Relative Lung Weight (RLW), and Survival Rate

The ABW from each group was recorded from week 0 and continuously monitored every 2 weeks for 22 weeks. The RLW was estimated using the formula below after sacrificing the animals from all groups, as described earlier [49]. The ABWs of the mice from each group were recorded every two weeks throughout the study. All the surviving animals after 22 weeks were monitored for 18 more weeks until the end of week 40, following the first exposure to the carcinogen (BaP).

(1) $$\mathrm{RLW}=\frac{Lungs\;weight}{Body\;weight}\times 100$$

### 2.8. Histopathological Evaluation of Lungs

The histopathological analysis of H&E-stained slides was conducted to investigate the chemopreventive potential of AAE. Briefly, the routine procedure was used for hematoxylin and eosin (H&E) staining following the processing of formalin-fixed tissues and sectioning. The photomicrographs of all the slides, taken under the light microscope at 100× and 400× magnifications with 100 µm and 50 µm scale bars, respectively, were then analyzed for all the treated groups.

### 2.9. Serum Biochemical Analyses

The activities of cancer marker enzymes, lactate dehydrogenase (LDH), adenosine deaminase (ADA), gamma glutamyl transferase (γ-GT), and 5′-nucleotidase (5′-NT) were analyzed in the serum of all treated groups. The kits from the Abcam were used for all these enzymes, and the protocols given in the respective kits by the manufacturer were followed.

### 2.10. Antioxidant Enzyme Assays in Lung Tissues

The levels of antioxidants were examined after homogenizing the lung tissues in the buffer, provided with the kits of the respective enzymes, for superoxide dismutase (SOD), catalase (CAT), malondehyde dehydrogenase (MDA), and glutathione peroxidase 1 (GPx1).

### 2.11. Annexin V-FITC/PI Apoptosis Assay

The annexin V-FITC and PI staining of the lung cells was analyzed for the distribution of cells in different quadrants, with live cells in the lower left, necrosis in the upper left, early apoptosis in the lower right, and late apoptosis in the upper-right quadrant, using FlowJo following the acquisition of the sample in the MACSQuant analyzer. Briefly, the single-cell suspension from the lungs was prepared for all experimental groups using the MACS tissue dissociator (Miltenyi Biotec, Bergisch Gladbach, Germany). The cells were suspended in binding buffer following filtration with a 100-µm mesh cell strainer and then centrifuged at 300× *g*. The samples were acquired in the MACSQuant analyzer 10 (Miltenyi Biotec, Germany) after incubating the cells for 20–25 min with the AnnexinV-FITC-PI provided in the kit. All the samples were analyzed, and the distributions of the cells were plotted by FlowJo software v10.8.1.

### 2.12. Determination of Cellular ROS Generation by Flow Cytometry

The 2′,7′-dichlorofluorescin diacetate (DCFDA) staining of the lung cells was analyzed as the mean fluorescence intensity (MFI) using FlowJo following the acquisition of the sample in the MACSQuant analyzer. The cells were suspended in the DMEM media following filtration with a 100 µm mesh cell strainer and then centrifuged at 300× *g*. The samples were acquired in the MACSQuant analyzer 10 (Miltenyi Biotec, Germany) after incubating the cells for 40 min at 37 °C with 20 µM DCFDA. All the samples were analyzed as the MFI of DCFDA, and histograms were plotted by the FlowJo software v10.8.1.

### 2.13. Statistical Analysis

Multiple comparisons were performed for all the experimental groups using the mean values and standard errors. The significant differences between the experimental groups were analyzed by one-way and two-way ANOVA and Tukey’s multiple comparison tests using Prism 9. A *p*-value < 0.05 was considered statistically significant.

## 3. Results

### 3.1. AAE Yield Percentage and Its Potent Antibacterial and Antifungal Activity

**Figure 2:**
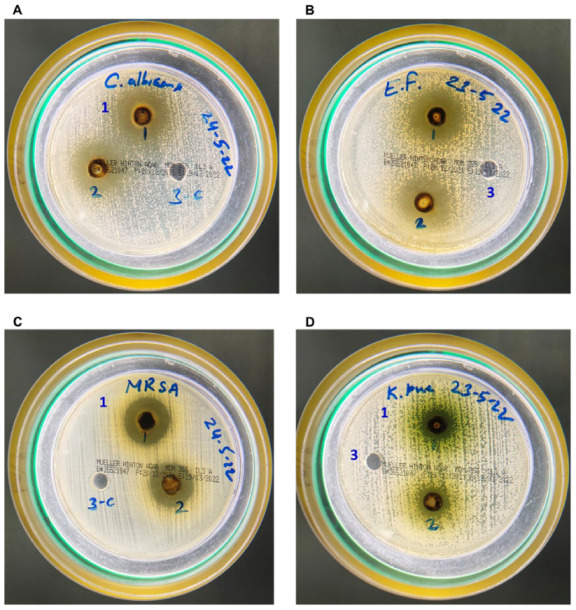
AAE shows antimicrobial activity against (**A**) *C. albicans*, (**B**) *E. faecalis*, (**C**) MRSA, and (**D**) *K. pneumoniae*.

**Figure 3:**
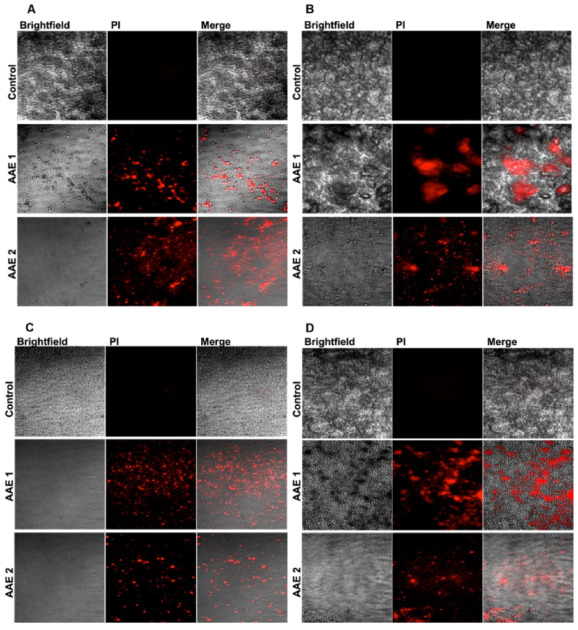
AAE demonstrates microbial death against (**A**) *C. albicans*, (**B**) *E. faecalis*, (**C**) MRSA, and (**D**) *K. pneumoniae*.

The AAE was weighed as 13.5 g after collecting the dried powder, so the yield of the extract was measured to be 27%. *C. albicans* showed remarkable susceptibility to AAE, as shown by the size of the zone of inhibition. There were zones of inhibitions of 24 mm (well no. 1) and 20 mm (well no. 2) around the wells loaded with 6 mg and 3 mg of AAE, respectively (Figure 2A and Table 1), whereas well no. 3, loaded with the control, did not show any inhibition in growth. Similarly, AAE also showed activity against both the Gram-positive and Gram-negative bacteria. Mueller Hilton Agar plates seeded with *E. faecalis* had 25 mm (well no. 1) and 21 mm (well no. 2) sizes of inhibition zones in wells loaded with 6 mg and 3 mg of AAE, respectively (Figure 2B and Table 1). Interestingly, AAE also demonstrated activity against MRSA, as well no. 1, loaded with 6 mg AAE, had a 21 mm inhibition zone, and well no. 2, loaded with 3 mg AAE, exhibited an 18 mm inhibition zone against MRSA (Figure 2C and Table 1). AAE also inhibited the growth of the Gram-negative bacterium *K. pneumoniae*. Well no. 1, loaded with 6 mg of AAE, showed an inhibition zone of 14 mm, whereas well no. 2, containing 3 mg of AAE, demonstrated a 12 mm inhibition zone against *K. pneumoniae* (Figure 2D and Table 1). Well no. 3, loaded with vehicle control, did not show an inhibition zone (Figure 2A–D). The data also demonstrated the cell death by AAE under confocal microscope, confirming its antimicrobial activity (Figure 3A–D). The MICs of AAE were found to be 400 µg/mL against *C. albicans* and *E. faecalis* and 500 µg/mL against MRSA, whereas the MIC (1 mg/mL) of AAE was found to be higher against *K. pneumoniae*.

### 3.2. Effect of AAE on BaP-Induced ABW, Survival, and RLW

**Figure 4:**
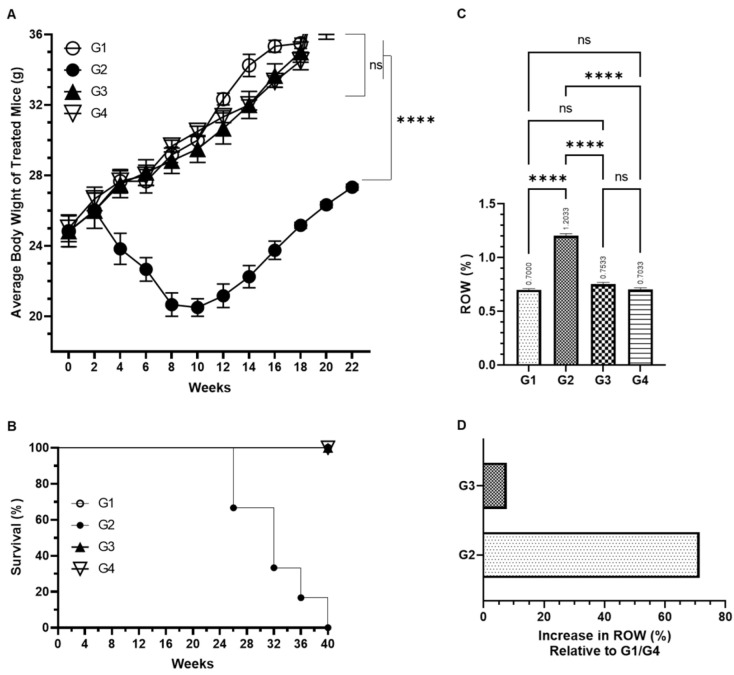
Effect of AAE on carcinogen-moderated (**A**) ABW, (**B**) survival, (**C**) RLW, and (**D**) increased percentage of RL relative to G1/G4. The values are given as the SEM of 5 animals in each group for (**A**,**C**), whereas it is ten mice for (**B**). *^ns^* No significance within the groups, **** Significant difference between the groups, *p*-value < 0.0001.

The results demonstrated significant change in the ABWs in the animals exposed to carcinogen in G2 after 22 weeks before sacrifice, as it was registered to be 27.33 g, while 37.16 g was measured in G1. As shown in Figure 4A, the ABW declined swiftly between week 4 and week 10, from 26.0 g to 20.5 g. However, ABW in G2 started increasing after this period but could not achieve the ABW of G1 or G4 (Figure 4A). The animals treated with AAE showed no significant changes in ABW after 22 weeks in G3 and were not affected by the exposure to BaP, as continued growths in the ABWs were monitored in these animals, as recorded in G1/G4. The Kaplan–Meir curve showed a 100% survival in the mice pretreated with AAE in G3, as the animals were monitored for up to 40 weeks for their survival, while it recorded a 60% mortality in BaP-only exposed G2 mice (Figure 4C). The data revealed a significant increase in ROW in the carcinogen exposed to the mice, as it was escalated to 1.2% in G2 (71.4% relative to G1/G4). However, AAE-pretreated mice showed significant resistance to BaP, as the ROW was estimated to be 0.75% in G3 mice (Figure 4D).

### 3.3. Effect of AAE on Cancer Marker Enzymes as ADA, AHH, GGT, 5-NT (CD73), and LDH in the Serum Induced by Carcinogen

**Figure 5:**
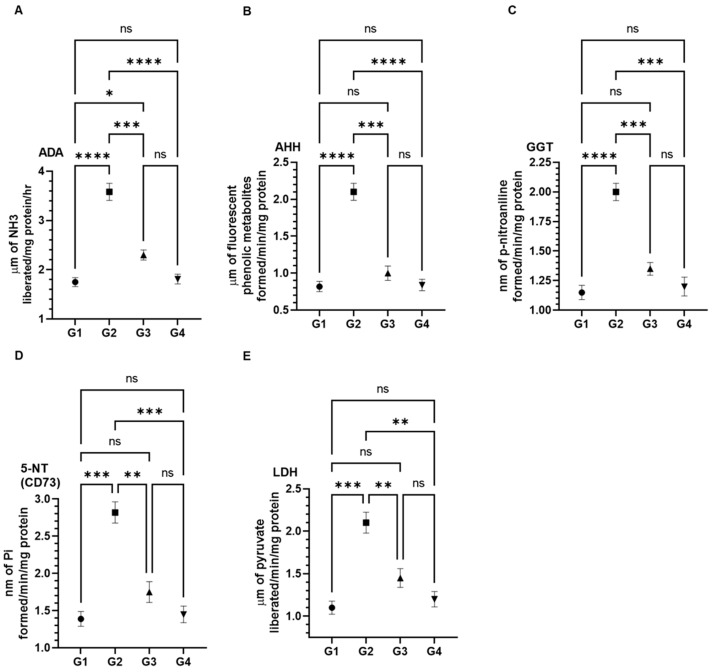
Effects of AAE on BaP-induced cancer marker enzymes in the serums (**A**) ADA, (**B**) AHH, (**C**) GGT, (**D**) 5-NT (CD73), and (**E**) LDH. The values are given as the SEM of three different experiments. *^ns^* No significance within the treated groups; * significant difference between the groups, *p*-value < 0.05; ** significant difference between the groups, *p*-value < 0.01; *** significant difference between the groups, *p*-value < 0.001; **** significant difference between the groups, *p*-value < 0.0001.

The results demonstrated a significant recovery in activities of cancer markers in the serum by AAE in G3 mice. As depicted in Figure 3, the exposure of BaP increased the activities as ADA (3.58 µm ± 0.17 SEM), AHH (2.1 µm ± 0.11 SEM), GGT (2.0 µm ± 0.07 SEM), CD73 (2.82 µm ± 0.14 SEM), and LDH (2.1 µm ± 0.12 SEM) (Figure 3). However, the level of these enzymes in the serum of G1 mice were recorded to be 1.75 µm ± 0.09 SEM (ADA), 0.82 µm ± 0.07 SEM (AHH), 1.15 µm ± 0.06 SEM (GGT), 1.39 µm ± 0.1 SEM (CD73), and 1.1 µm ± 0.08 SEM (LDH). Interestingly, significant drops in the activities of these cancer markers were estimated to be close to normal levels: 2.3 µm ± 0.1 SEM (ADA), 1.0 µm ± 0.1 SEM (AHH), 1.35 µm ± 0.05 SEM, 1.75 µm ± 0.14 SEM (CD73), and 1.45 µm ± 0.11 SEM (LDH). Noticeably, no changes were measured in the activities of these enzymes in G4 while comparing it to G1 (Figure 5).

### 3.4. Effect of AAE on Carcinogen-Modulated Antioxidant Enzymes in Lung Tissues

**Figure 6:**
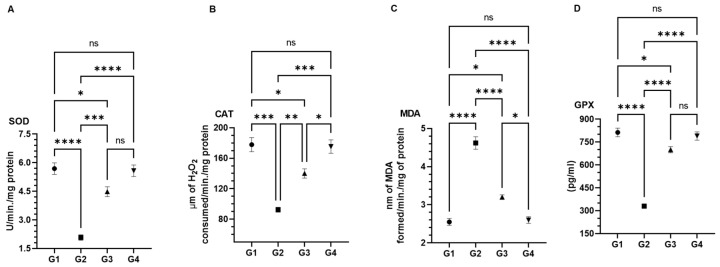
Effect of AAE on antioxidant enzymes in lung tissues: (**A**) SOD, (**B**) CAT, (**C**) MAD, and (**D**) GPx1. The values are given as the SEM of three different experiments. *^ns^* No significance within the groups; * significant difference between the groups, *p*-value < 0.05; ** significant difference within the groups, *p*-value < 0.01; *** significant within the groups, *p*-value < 0.001; **** significant difference within the groups, *p*-value < 0.0001.

The data demonstrated the significant recovery of SOD, CAT, MDA, and GPx1 in AAE-pretreated G3 mice that were significantly modulated by their exposure to BaP in G2 (Figure 6). As shown in Figure 4, the significant rise was estimated as 4.48 U ± 0.26 SEM (SOD), 140 µm and ± 6.1 SEM (CAT), and 3.2 pg ± 0.1 SEM (GPx) in AAE-pretreated G3 mice; that decreased to 2.018 U ± 0.14 SEM (SOD), 92.5 µm ± 3.2 SEM, and 330.5 pg ± 13.8 SEM by BaP in G2 mice. The levels of SOD, CAT, and GPx1 were measured as 5.69 U ± 0.30 SEM, 177.8 µm ± 9.1 SEM, and 813.25 pg ± 28.10 SEM, respectively, in vehicle-treated G1 mice. A significant upsurge in the level of MDA was recorded as 4.62 nm ± 0.16 SEM in BaP-exposed G2 mice, compared to 2.5 nm ± 0.09 SEM in G1 mice. However, the pretreatment of AAE in G3 significantly reduced the level of MDA to 3.2 nm ± 0.1 SEM. Noticeably, no changes were observed in the levels of these antioxidant enzymes in AAE-only-treated G4 mice, compared to the vehicle-treated G1 mice (Figure 6).

### 3.5. Effect of AAE on Cellular ROS in the Lung Cells by DCFDA Using Flow Cytometry

**Figure 7:**
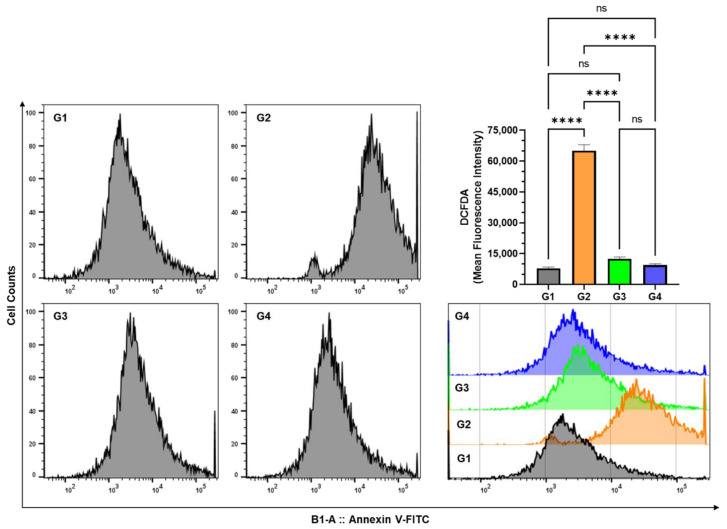
Effect of AAE on cellular ROS in the lung cells by DCFDA using flow cytometry. The values are given as the SEM of three independent experiments. *^ns^* No significance within the groups; **** significant difference between the groups, *p*-value < 0.0001.

The DCFDA staining of the lung cells was analyzed with the mean fluorescence intensity (MFI) using FlowJo following the acquisition of the sample in the MACSQuant analyzer. The results showed a significant induction of cellular ROS in BaP-exposed G2 mice, as its MFI reached 65100 ± 2888.5 SEM, while it was measured as 7833.3 ± 643.8 SEM and 9466.7 ± 606.5 SEM in G1 and G4, respectively (Figure 7). However, AAE showed chemopreventive potential and significantly reduced the cellular ROS, as it recorded a 12,500 MFI ± 866.025 SEM in G3 mice (Figure 7).

### 3.6. Effect of AAE on the Induction of Apoptosis in the Lung Cells by Annexin V-FITC-PI Using Flow Cytometry

**Figure 8:**
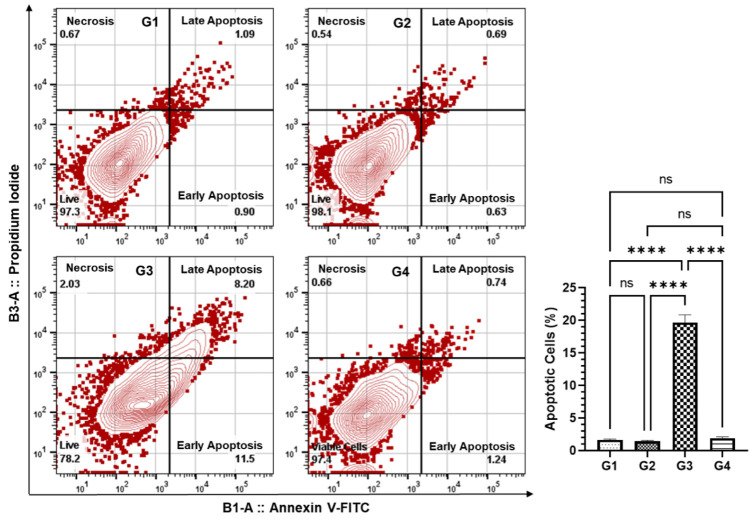
Effect of AAE on the induction of apoptosis in the lung cells by Annexin V-FITC-PI using flow cytometry. The values are given as the SEM of three different experiments. *^ns^* No significance within the groups; **** significant difference between the groups, *p*-value < 0.0001.

The annexin V-FITC and PI staining of the lung cells was analyzed using FlowJo following the acquisition of the sample in the MACSQuant analyzer. The analysis showed that 36% of the cells were induced to apoptosis in G3 mice pretreated with AAE, while no induction of programmed cell death was measured in any other group (Figure 8).

### 3.7. Effect of AAE on the Histopathology of BaP-Modulated Lungs

**Figure 9:**
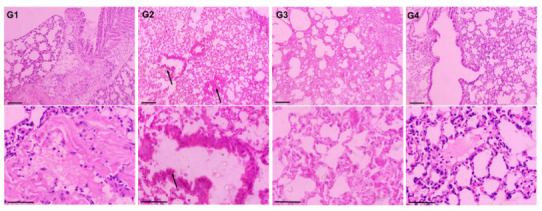
Effect of AAE on BaP-mediated carcinogenesis in lungs by histopathological studies. UP, 100× magnification, bar = 100 µm; LP, 400× magnification, bar = 50 µm.

The H&E staining of the lungs from all the treated groups revealed the chemopreventive potential of AAE against lung carcinoma induced by BaP (Figure 9). The representative images from all the groups were arranged, with the upper panel (UP) shown at a 100× magnification with a 100 µm scale bar and the lower panel (LP) shown at a 400× magnification with a 400 µm scale bar. The H&E staining revealed the damages in the alveoli and the clusters of hyperchromatic cells, which had irregular nuclei and scant cytoplasm with adjacent alveolar hyperplasia in the lungs of the G2 mice exposed only to BaP. However, the lungs from the G3 mice pretreated with AAE showed normal alveolar structures and respiratory bronchioles, similar to G1/G3 (Figure 9).

## 4. Discussions

The *A. annua* and its active constituents, especially artemisinin, have shown antimicrobial and anticancer activity in vitro, as well as in vivo. The antimicrobial activity of the AAE was tested against four microbes as one fungal and three bacterial strains. The data revealed the great sensitivity of AAE against *C. albicans,* as it showed a 20 mm zone of inhibition (Figure 3A and Table 1), and more than 50% of the cells were dead (Figure 3A) in 3 mg/mL. However, Gala et al. 2005 reported no effects of artemisinin against *C. albicans* and *Cryptococcus neoformans* [50]. AAE is the mixture of several constituents that could have the potential due to the synergistic effect of these secondary metabolites instead of only artemisinin. Earlier, the effect of essential oil from the aerial parts of *A. annua* was reported against *E. faecalis* [21]. The results displayed the zone of inhibition as 21 mm (Figure 2B and Table 1), with less than 50% of viable cells (Figure 3B) against *E. faecalis* treated with a lower concentration of AAE. The AAE also demonstrated antimicrobial potential against the Gram-positive *K. pneumoniae,* with a 12 mm zone of inhibition, and ~50% of the cells were recorded dead in 3 mg/mL of the extract (Figure 2D and Figure 3D). This is the first study to evaluate the efficacy of *A. annua* against *K. pneumoniae*. Interestingly, the data also suggested the bactericidal potential of AAE against MRSA, as a 21 mm zone of inhibition (Figure 2C and Table 1) was found, and more than 90% of the cells (Figure 2C) were dead when treated with 6 mg/mL. Earlier, some studies reported the efficacy of *A. turanica* and *A. dracunculus* against MRSA in vitro and in vivo respectively [51,52]. Previous studies suggested the antimicrobial potential of plant extracts against MRSA due to the presence of the compounds having β-lactamase inhibiting properties [53,54].

The potential of the extract of *A. annua* has been reported to inhibit the cellular proliferation of MDA-MB-231 and MCF-7 breast cancer, PC-3 prostate cancer, and MIA PaCa-2 pancreas cancer cells. The *antitumor* activity of the extract of *A. annua* was evaluated against a triple negative breast cancer xenograft in nude mice [22]. This was also touched upon by Rassias and Weathers, who exhibited the efficacy of the dried leaf extract of Artemisia against A549, H1299, and PC9 non-small-cell lung cancer (NSCLC). They also demonstrated the potential of the extract in the inhibition of tumor growth against A549- and PC9-implanted tumor xenograft models [33]. The present study demonstrated the in vitro antimicrobial activity and in vivo chemopreventive potential of AAE in a carcinogen-induced lung cancer model in detail.

Several studies have reported a significant drop in body weight due to the cancer cachexia, which was also observed in the animals exposed to BaP for 4 to 10 weeks (Figure 4A) [55,56]. AAE showed the prevention of cancer cachexia, as no changes in the body weights were measured in G3 mice. Multiple studies have demonstrated that the excess of inflammatory cells and immortal proliferation of malignant cells affect the size of the lungs, which were enlarged [48,57]. The data revealed that the BaP-only exposure increased ROW to 71.5% in G2 mice (1.2%), compared to G1/G4 (0.7%). However, AAE prevented the significant increase in ROW, as only 0.75% was noticed in G3 mice.

The changes in the activities of various cancer markers are also suggested to be evaluated in carcinogen-modulated tumorigenesis [58,59,60,61]. The biochemical analyses of AHH, γ-GT, 5′-NT, ADA, and LDH in the serum exhibited significant drops of these enzymes to the normal levels by AAE in G3; they were measured to be higher in the BaP-exposed G2 mice. The data revealed the role of AAE in the prevention of lung cancer promotion and progression, following the delay in the initiation of carcinogenesis (Figure 5).

The induction of carcinogenesis, followed by the promotion and progression by polycyclic hydrocarbons such as BaP in the lung cancer animal model, is comparable to the event of carcinogenesis in the lungs by environmental pollutants. The generation of ROS during this process plays the significant role in BaP-induced lung carcinoma, as the lungs are directly exposed to it through oral gavage [62,63,64]. As depicted in Figure 6C, AAE significantly prevented the release of MDA, which is the product of lipid peroxidation, clearly indicating its potential to protect the lungs from cancer promotion. The H&E staining of lung tissues confirmed this efficacy, as no structural changes were observed in AAE-pretreated G3 mice (Figure 9). Evidently, the antioxidants have nutritional and health benefits, while there is an inverse correlation between them and the ROS. Therefore, the antioxidants are said to have been overwhelmed by the ROS. Interestingly, the antioxidants have various levels of defense that could be radical prevention, radical scavenging, as well as radical-induced damage repair. Due to these different layers of defense, the antioxidants are characterized as the first- to fourth-lines-of-defense antioxidants. The first-line-of-defense antioxidants actively neutralize the molecule, which has the potential to generate the ROS or free radicals. In this line of defense, SOD, CAT, and GPX are considered the main enzymes that participate to avoid the generation of ROS [65]. The modulation in the activities of these antioxidants to the exposure of chemical carcinogens, including BaP, has been demonstrated in several studies [66,67,68,69]. As evident from the results, AAE protected the reduction in the activity of these enzymes in G3 lungs, while they were estimated to be significantly low in G2, compared to G1/G4 (Figure 6). In addition, the flow cytometry analysis data also confirmed the significant retrieval of antioxidants, as the DCFDA significantly decreased to 12,500 MFI from 65,100 MFI of the BaP-exposed G2, while the G1 and G4 showed 7833 and 9466 MFI, respectively (Figure 7). Subsequently, the frequency of apoptosis was evaluated by flow cytometry, which showed that 20% of the cells in the lung cells of G3 mice were apoptotic. The apoptotic data also confirmed the chemopreventive potential of AAE in the exclusion of abnormal cells through programmed cell death to maintain the tissue homeostasis (Figure 8).

## 5. Conclusions

The current findings demonstrated the use of AAE as an alternative medicine in the treatment of infectious disease and the chemoprevention of lung cancer. To our knowledge, this is the first study that summarizes the chemopreventive potential of AAE in a lung cancer model in vivo. The pre-treatment of AAE not only protected the lungs from the development of SCLC but also significantly inhibited the cellular proliferation of abnormal cells by inducing programmed cell death. However, further investigations are suggested to understand the role of AAE to potentiate the therapeutic index of commercially available of the drugs that show multiple drug resistance and/or high toxicity in detail.

## Figures and Tables

**Table 1:** Measurements of the zones of inhibition of the antimicrobial activity of AAE against *C. albicans*, *E. faecalis*, MRSA, and *K. pneumoniae*.

Microorganism	Zone of Inhibition MEA (6 mg)	Zone of Inhibition MEA (3 mg)
*Candida albicans*	24 mm	20 mm
*Enterococcus faecalis*	25 mm	21 mm
MRSA	21 mm	18 mm
*Klebsiella pneumoniae*	14 mm	12 mm

## Data Availability

Not applicable.
